# Thinking outside the cavity: Effusion lymphoma primary to bone marrow

**DOI:** 10.1002/ccr3.4100

**Published:** 2021-05-20

**Authors:** Sean X. Gu, Zenggang Pan, Mina L. Xu

**Affiliations:** ^1^ Department of Laboratory Medicine Yale School of Medicine New Haven CT USA; ^2^ Department of Pathology Yale School of Medicine New Haven CT USA

**Keywords:** AIDS‐related lymphoma, extracavitary, primary effusion lymphoma

## Abstract

This case illustrates a rare and aggressive entity in AIDS‐related lymphoproliferative disorders and highlights the importance and challenges of recognizing PEL outside of cavitary lesions.

## Graphical abstract

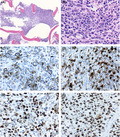

## 1 CASE DESCRIPTION

Primary effusion lymphoma (PEL) is a distinct disease entity of large B‐cell lymphomas most often occurring in immunocompromised patients. We present a rare case of extracavitary PEL primary to the bone marrow in a HIV‐positive patient.

**FIGURE 1:**
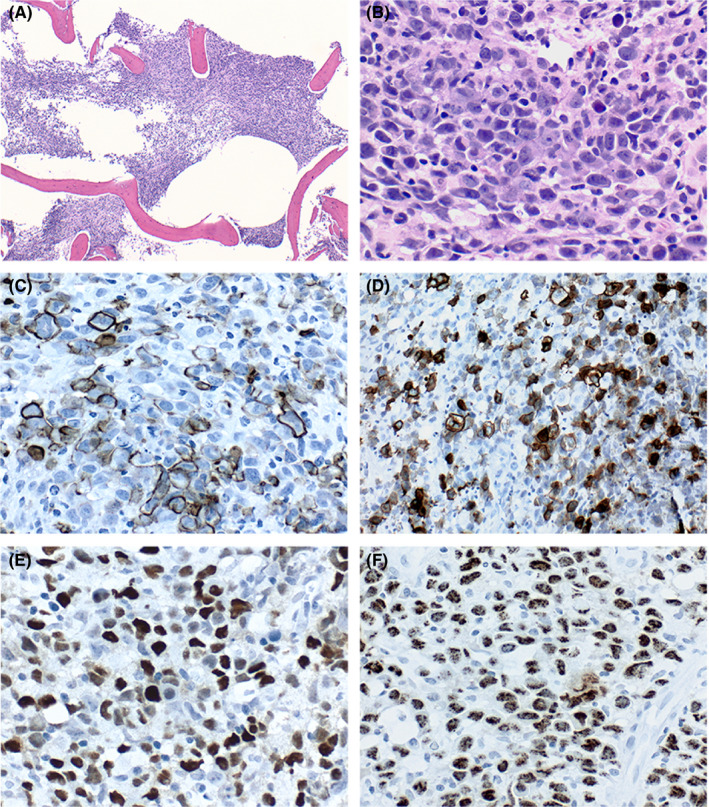
Bone marrow biopsy core histology (hematoxylin and eosin, original magnifications ×4 (A), ×40 (B). Immunohistochemistry for CD138 (C), CD30 (D), in situ hybridization for EBER (E) and HHV‐8 (F)

A 41‐year‐old HIV‐positive man presented to the emergency department with fever, malaise, and dyspnea. Splenomegaly was noted on physical examination but no lymphadenopathy or cavitary effusions. His viral load was markedly elevated with CD4 count of 16 cells/µL, and he was pancytopenic. Clinical suspicion was high for infection, acute leukemia, or hemophagocytic lymphohistiocytosis. Bone marrow biopsy showed sheets of large atypical mononuclear cells with open chromatin, distinct nucleoli, and ample basophilic cytoplasm, compatible with plasmablastic morphology (Figure 1A,B). Tumor cells were positive for CD138 (Figure 1C) and CD30 (Figure 1D) while negative for CD45, CD3, CD20, PAX5, OCT2, and CD79a. Differential diagnoses included plasmablastic lymphoma, plasma cell myeloma with plasmablastic features, and primary effusion lymphoma (PEL). Both in situ hybridization for Epstein‐Barr virus (EBV) (Figure 1E) and immunohistochemical staining for human herpesvirus‐8 (HHV‐8) (Figure 1F) were positive in tumor cells; hence, the diagnosis of extracavitary PEL was rendered. Chemotherapy was initiated; nevertheless, the patient died of disease 19 days after diagnosis.

Primary effusion lymphoma is a distinct clinicopathologic entity of AIDS‐related lymphomas. Classic cavitary PEL is characterized by malignant effusions with plasmablastic lymphoid cells expressing plasma cell‐related markers. While EBV infection is common, the presence of HHV‐8 (ie, Kaposi's sarcoma‐associated herpesvirus) in the nuclei is essential to the diagnosis. Extracavitary PEL is a rare clinical variant of PEL that presents with solid tumor lesions in the absence of malignant serous effusions.^1^, ^2^ This case highlights the clinical and diagnostic features of PEL, and the importance of recognizing PEL outside of cavitary lesions.

## CONFLICT OF INTEREST

None declared.

## AUTHOR CONTRIBUTIONS

SXG: collected patient information and wrote the manuscript. ZP: provided support for photomicrographs and helped revise the manuscript. MLX: provided insights and comments and helped revise the manuscript. All authors: approved the final version.

## ETHICAL APPROVAL

The manuscript was prepared according to standard publication ethical guidelines.

## INFORMED CONSENT

Informed consent has been obtained from the patient.

## Data Availability

The authors have no relevant funding sources to report and any relevant data will be made available and accessible.
